# A High Temperature Capacitive Pressure Sensor Based on Alumina Ceramic for *in Situ* Measurement at 600 °C

**DOI:** 10.3390/s140202417

**Published:** 2014-01-30

**Authors:** Qiulin Tan, Chen Li, Jijun Xiong, Pinggang Jia, Wendong Zhang, Jun Liu, Chenyang Xue, Yingping Hong, Zhong Ren, Tao Luo

**Affiliations:** 1 Key Laboratory of Instrumentation Science & Dynamic Measurement, Ministry of Education, North University of China, Tai Yuan 030051, China; E-Mails: tanqiulin.99@163.com (Q.T.); jpg021@163.com (P.J.); wdzhang@nuc.edu.cn (W.Z.); liuj@nuc.edu.cn (J.L.); xuechenyang@nuc.edu.cn (X.C.); 18618256601@163.com (Y.H.); flanklc1987@163.com (Z.R.); luotaonuc@163.com (L.T.); 2 Science and Technology on Electronic Test & Measurement Laboratory, North University of China, Tai Yuan 030051, China

**Keywords:** high-temperature pressure sensor, *in situ* test, alumina ceramic, LC circuit

## Abstract

In response to the growing demand for in situ measurement of pressure in high-temperature environments, a high temperature capacitive pressure sensor is presented in this paper. A high-temperature ceramic material-alumina is used for the fabrication of the sensor, and the prototype sensor consists of an inductance, a variable capacitance, and a sealed cavity integrated in the alumina ceramic substrate using a thick-film integrated technology. The experimental results show that the proposed sensor has stability at 850 °C for more than 20 min. The characterization in high-temperature and pressure environments successfully demonstrated sensing capabilities for pressure from 1 to 5 bar up to 600 °C, limited by the sensor test setup. At 600 °C, the sensor achieves a linear characteristic response, and the repeatability error, hysteresis error and zero-point drift of the sensor are 8.3%, 5.05% and 1%, respectively.

## 1. Introduction

The precise measurement of pressure in high-temperature environments is critical in many applications such as in the automotive industry, aerospace, aeronautics, advanced industry, aero-engine turbines, and the civil industry [1–4]. The sensors used for these applications are required to work in high-temperature environments, at temperatures ranging from 400 °C to 800 °C. Despite the successful development of many pressure sensors relying on piezoresistance for dynamic pressure monitoring, these sensors are based on silicon and cannot operate in higher-temperature environments because the leakage current across the junctions changes drastically above 150 °C and the mechanical properties easily deteriorate with increasing temperature and pressure [5,6]. Sensors based on Silicon-On-Insulator (SOI) technology can work in higher temperature environments when compared with silicon sensors with PN junctions, but the sensors become invalid at 500 °C [7,8]. To date, some pressure sensors based on ceramics have been developed, but their performance is poor. For example, a high-temperature pressure sensor was designed and fabricated by the Georgia Institute of Technology, based on Low-Temperature Co-Fired Ceramic (LTCC) material, but it was only tested up to 450 °C [9–12]. In 2013, Xiong *et al.* designed two sensors based on two different types of ceramic materials—a LTCC-based capacitance pressure sensor and a High-Temperature Co-Fired Ceramic (HTCC)-based capacitance pressure sensor. The performance of these sensors is better than that of the aforementioned sensors, but, they can't be operated above 600 °C [13,14]. Recently, Tan *et al.* also fabricated a pressure sensor using HTCC MEMS technology for use in harsh environments. This sensor can operate in high-temperature environments, but the coupling distance is only 2.8 cm at room temperature and the coupling strength will weaken quickly as the temperature increase [15]. In addition, the abovementioned ceramic sensors are wireless passive capacitive ceramic pressure sensors, which capture pressure signals through mutual inductance coupling with the antenna. The sensor working in high-temperature environments and the reader antenna working in low-temperature environments can be realized through the wireless passive coupling test method, however, the coupling effect becomes very weak in higher temperature environments, and testing the peak resonance frequency and the actual secondary circuit resonance frequency has a certain deviation, which is not conducive to the signal collection [11].

**Figure 1.**
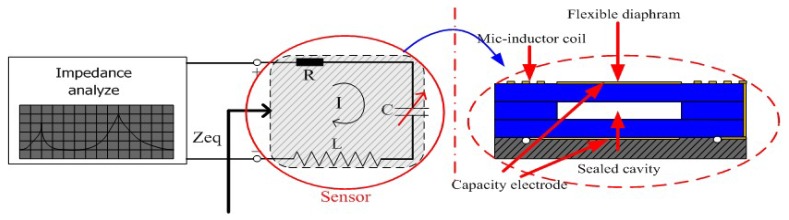
(**Left**) Pressure testing schematic; (**Right**) Design schematics of the sensor.

In order to solve the problem wherein wireless passive pressure sensors capture pressure signals with difficulty in high-temperature environments, the authors have proposed a sensor based on an alumina ceramic. Alumina is a high-temperature ceramic and has stable electrical properties and mechanical robustness in high-temperature environments. In addition, the proposed sensor is not a wireless passive pressure sensor, and signal collection is performed by supplying power to the sensor, as shown in Figure 1. Further, the design method realizes pressure parameter sensing by monitoring of the resonant frequency variation caused by the capacitance change. The inductor and variable capacitance are integrated in the alumina ceramic substrate through a thick-film integrated technology to complete the sensor fabrication. The high-temperature characterization of the fabricated sensor will be tested in a high-temperature sintering furnace from room temperature to 850 °C to verify the performance of the sensor in high-temperature environments. Finally, the achieved sensor was tested to realize pressure testing between atmospheric pressure and 5 bar in a high-temperature pressure test setup in the range from room temperature to 600 °C to demonstrate the pressure sensing capabilities of the sensor in high-temperature environments.

## 2. Model Analysis and Structure Design

The schematic of the pressure sensing system is shown in Figure 1. From Figure 1, the sensor is designed to have a constant inductance and a variable capacitive, and the capacitive reactance and inductive reactance change with increasing of the working frequency. Therefore, the input impedance of the series resonance circuit changes with the variation of the working frequency. In addition, the equivalent impedance *Z*_eq_ of the sensor is defined as:

(1) Z(jw)eq=R+j(wl−1wc)φ=arctanwl−1wcR

where *R* is the resistance of the sensor, *w* is the angular frequency of the signal source, *l* is the inductance value of the sensor, and *c* is the capacitance value of the sensor.

From Equation (1), it can be seen that the impedance phase is equal to 0 when the inductive reactance of the inductance is equal to the capacitive reactance of the capacitance. Therefore, a minimum occurs at the resonant frequency. In addition, when the excitation frequency is equal to the resonance frequency of the LC series resonance circuit, the series resonant circuit impedance is equal to *R*. Further, when the excitation current flows through the sensor, the phase and amplitude of the equivalent impedance of the sensor are expressed as follows:

(2) |Zeq|=Re2{Zeq}+Im2{Zeq}=R∠Zeq=arctanIm{Zeq}Re{Zeq}=0

It can be concluded that during the magnitude change to the minimum, the phase is equal to 0, when the excited frequency is equal to the resonance frequency of the sensor. As shown in Figure 1, the sensor is designed to have an electrical LC series resonance circuit, which consists of a constant inductor *L* and a variable capacitance *C*. In addition, the classical expression for the resonant frequency *f* can be represented as:

(3) $$f=\frac{1}{2\pi }\sqrt{\frac{1}{LC}-\frac{R^{2}}{L^{2}}}≅\frac{1}{2\pi \sqrt{LC}}\;\text{if}\;R\ll \sqrt{\frac{L}{C}}$$

where *R* denotes the resistance of the sensor. The variation in the capacitance leads to a change in the resonant frequency. Therefore, the measurement of the pressure variation is translated into that of the sensor's resonant frequency shift *f*. The inductance of the planar spiral coil is calculated as [11]:

(4) $$L=2.96\times 10^{-6}\frac{n^{2}(\frac{d_{\text{out}}+d_{in}}{2})}{1+2.75(\frac{d_{\text{out}}-d_{in}}{d_{\text{out}}+d_{in}})}$$

where *n* is the number of turns of the inductor coil, *d*_in_ is the inner diameter, and *d*_out_ is the outer diameter.

**Figure 2.**
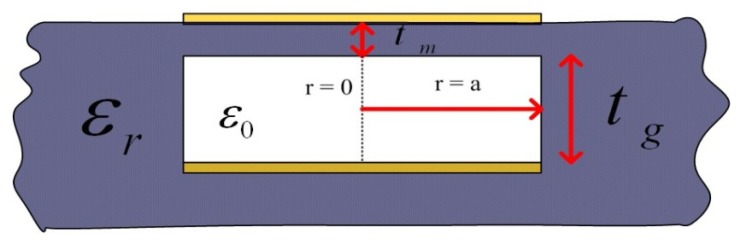
Cross-section of variable capacitance structure.

In this work, the change of single-layer sensitive membrane for the variable capacitance pressure sensor design is considered, as shown in Figure 2. When the air pressure inside the sealed cavity is different from that outside, elastic deformation of the ceramic sensitive membrane occurs. As the pressure increases the relationship between sensitive membrane deflection and pressure can be expressed as follows:

(5) $$d_{0}=\frac{3Pa^{4}(1-v^{2})}{16E{\left(t_{m}\right)}^{3}}$$

where *E* is the Young's modulus, *a* is the radius of the cavity, *v* is the Poisson's ratio, *t*_m_ is the thickness of the capacitance sensitive membrane. The change in pressure translates into change in capacitance, which is caused by the sensitive membrane change. In addition, a model that includes the deflection of the sensitive membrane is used for estimating the change in capacitance. The equation for the capacitance in pressure can be simplified as follows:

(6) $$Cs=\frac{{ɛ}_{0}\pi a^{2}}{t_{g}+\frac{2t_{m}}{{ɛ}_{r}}}\cdot \frac{\tanh^{-1}(\sqrt{\frac{0.00126Pa^{4}\times 12(1-0.0576)}{380\times 10^{9}{\left(t_{m}\right)}^{3}\cdot (t_{g}+\frac{t_{m}}{{ɛ}_{r}})}}}{\sqrt{\frac{0.00126Pa^{4}\times 12(1-0.0576)}{380\times 10^{9}{\left(t_{m}\right)}^{3}\cdot (t_{g}+\frac{t_{m}}{{ɛ}_{r}})}}}$$

where *a* is the radius of the electrode, *t*_g_ is the depth of the cavity, and *ε*_0_, *ε*_r_ are the free-space permittivity and relative dielectric constant, respectively.

In terms of the above sensor design realization, the sensor is predetermined with a sealed cavity to provide pressure reference in pressure sensing. The specific geometrical structure parameters of the inductor and capacitor are summarized in Table 1.

## 3. Fabrication

**Figure 3.**
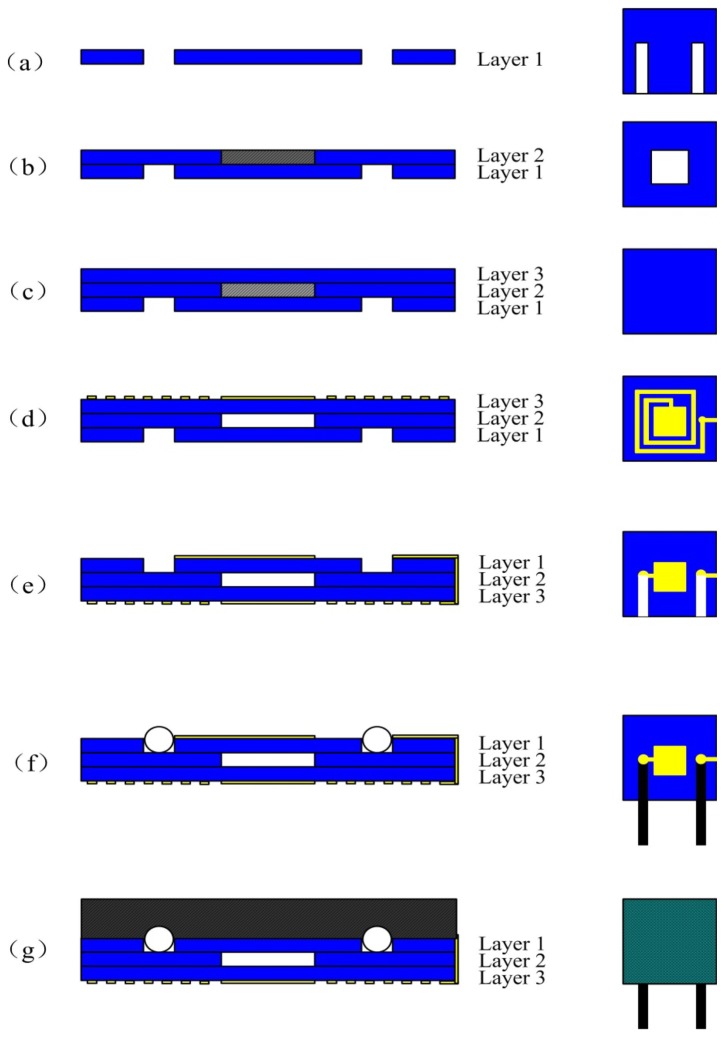
Fabrication process of the sensor.

**Figure 4.**
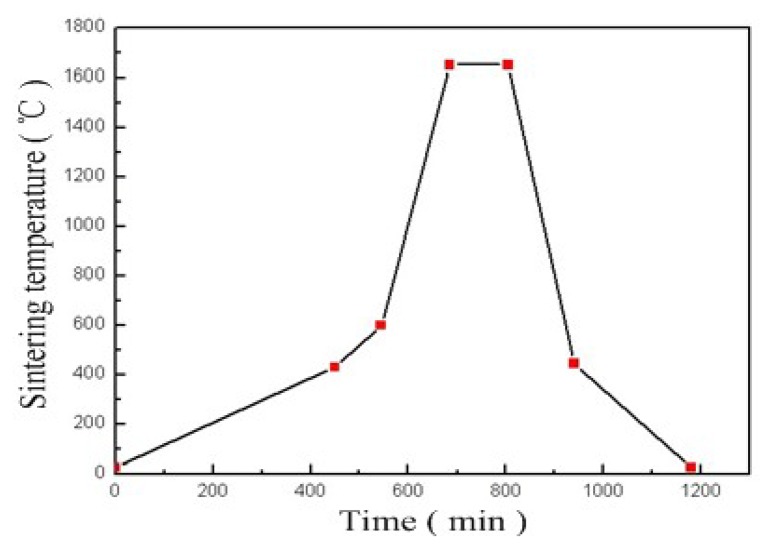
Ceramic substrate curing sintering curve.

**Figure 5.**
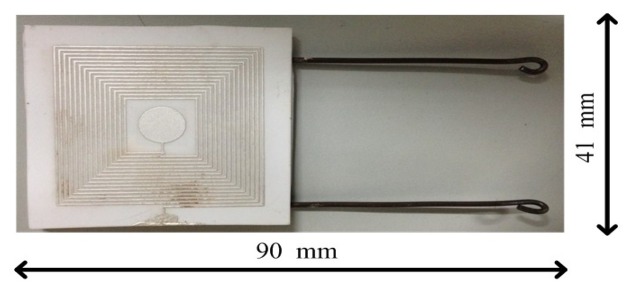
Photograph of the LC capacitance pressure sensor.

95% alumina ceramic layers (The Thirteenth Research Institute of Electronics Technology Group Corporation, Shijiazhuang, China) and Dupont Ag 6142D paste (DuPont, Wilmington, DE, USA) were the structural materials used to fabricate the sensor. The three ceramic layers form the sealed cavity and sensitive membrane through the multilayer ceramic substrate technology. In addition, the Dupont Ag 6142D electrode paste forms the capacitance plate and the inductance coil through a silk screen printing process. The fabrication process of the sensor is shown in Figure 3. The fabrication process started with pretreatment of the green tapes in a drying oven at 80 °C for approximately 30 min. A green tape was selected as layer 1 and cut for the channel using the NDYAG micro-machining laser system (Rofin, Standard, UK) as shown in Figure 3a. The next step was to cut the cavity with the designed punch file on layer 2 and the cavity was filled with a carbon film, as shown in Figure 3b. Following the previous fabrication step, layer 3 was laminated with layer 1 and layer 2, as shown in Figure 3c. The final stack was sintered in the box furnace at a peak temperature of 1,630 °C for 125 min with a total firing time of 20 h, as shown in Figure 4. The carbon film turned into CO_2_ during the sintering process and spreads to air through the ceramic substrate at a temperature of up to 500 °C, leading to the formation of the sealed cavity. After the sintering process, the Dupont Ag 6142D electrode paste was screen-printed on layer 1 for the capacitance plate and the inductance coil, and it was dried in an oven at 120 °C for 15 min, as shown in Figure 3d. Further, layer 3 was screened with the same silver paste for the wires and capacitance plate, as shown in Figure 3e. As shown in Figure 3f, a tungsten wire was placed as the electric wire in the channel and connected with the pads on layer 3. After the above process, the sensor was stinted in a seven-zone belt furnace at a peak temperature of 850 °C with a total firing cycle time of 70 min, which was used for silver paste curing. The speed of the belt is 100 mm/min, the time for each zone is approximately 10 min, and the specific sintering process parameters of the silver paste are listed in Table 2. Finally, the sensor is encapsulated with a high-temperature-resistant material to increase the stability of the components, and the completed sensor sample structure is shown in Figure 5. The outer dimensions of the fabricated sensor are approximately 90 mm × 41 mm × 3 mm.

## 4. Experiments and Discussion of the Results

The sensor was tested in high-temperature environments and high-thermal-stress environments. During testing, an impedance analyzer E4991A was used to extract the electrical parameters of the sensor, such as the inductance, capacitance, resistance, resonant frequency, and quality factor Q of the sensor. Table 3 lists the measured electrical parameters of the sensor in air.

### 4.1. High-Temperature Environments Characterization

**Figure 6.**
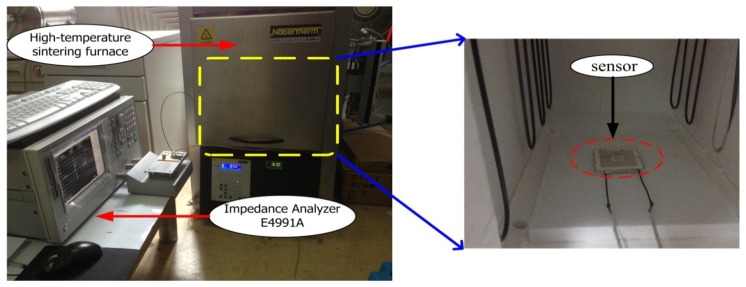
High-temperature measurement setup.

The high-temperature properties of the sensor are tested using a high-temperature measurement setup, which consist of an impedance analyzer and a high-temperature sintering furnace. The specific high-temperature measurement setup is shown in Figure 6. The temperature can be controlled accurately from room temperature up to 1,200 °C using the high-temperature sintering furnace. Further, the sensor was placed in the high-temperature sintering furnace to complete the high-temperature performance test in high-temperature environments. The resonant frequency of the sensor was measured by an E4991A impedance analyzer (Agilent/HP, Santa Clara, CA, USA) as a function of the temperature from room temperature to 850 °C.

**Figure 7.**
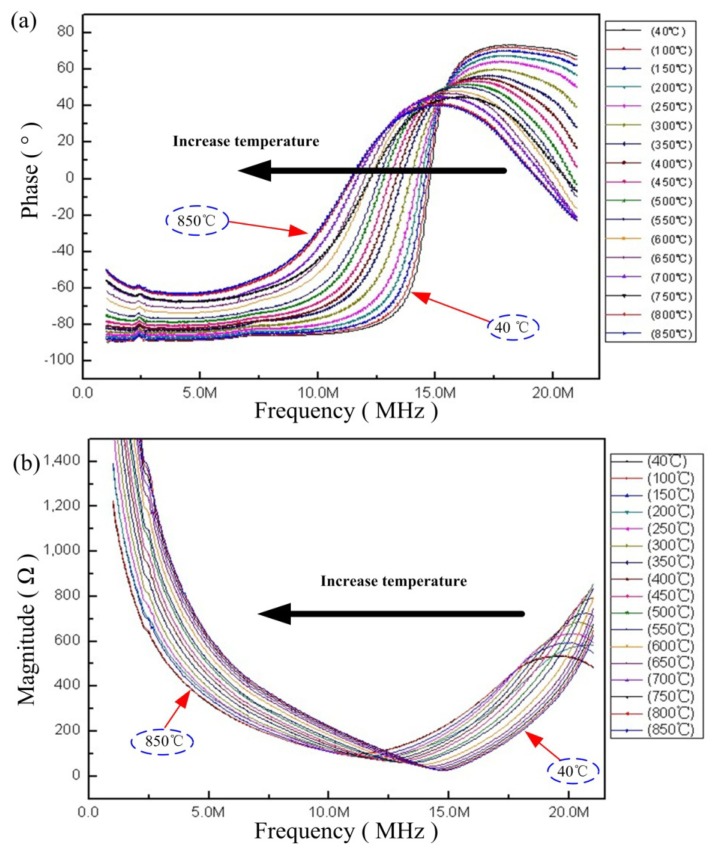
Measured impedance phase (**a**) and impedance magnitude (**b**) *versus* sensor frequency from 40 °C to 600 °C.

**Figure 8.**
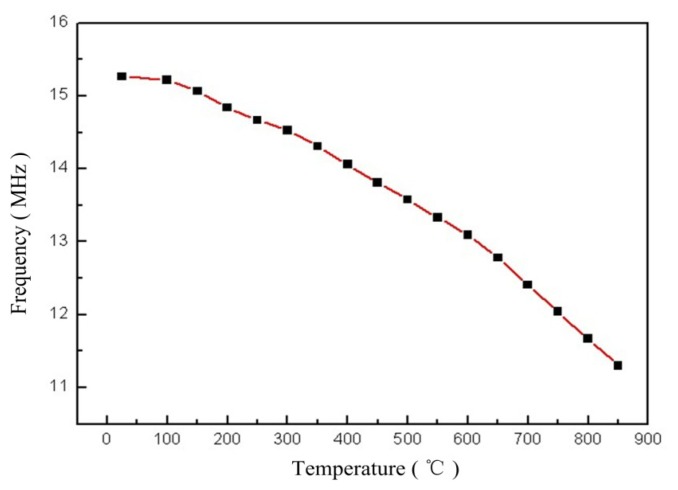
The resonant frequency of the sensor *versus* temperature.

**Figure 9.**
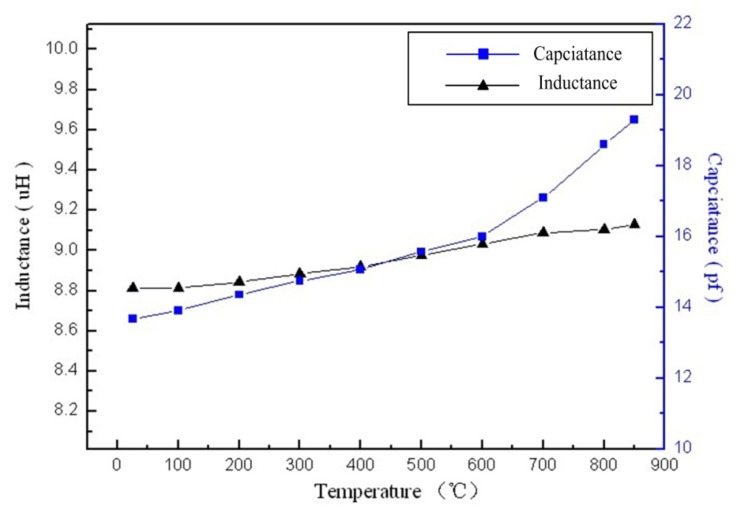
Change of inductance L and variable capacitance Cs *versus* temperature for the sensor.

Many high-temperature studies had been conducted to investigate the stability and performance of the sensor. The equivalent impedance phase and magnitude of the sensor were measured as functions of temperature from room temperature to 850 °C at atmospheric pressure, as shown in Figure 7. It is clear that the impedance phase and amplitude of the sensor can be detected easily. The resonant frequency of the sensor drifts evenly as the temperature increases uniformly, and the average slope is approximately −4.3 kHz·°C^−1^ from room temperature to 850 °C, as shown in Figure 8. From Figure 9, the measurement results show that the inductance and capacitance of sensor increase as the temperature increase, but the inductance of sensor change slight as the temperature increase. Therefore, the variation of capacitance and inductance of the sensor will influence the resonant frequency of the sensor as the temperature increases.

**Figure 10.**
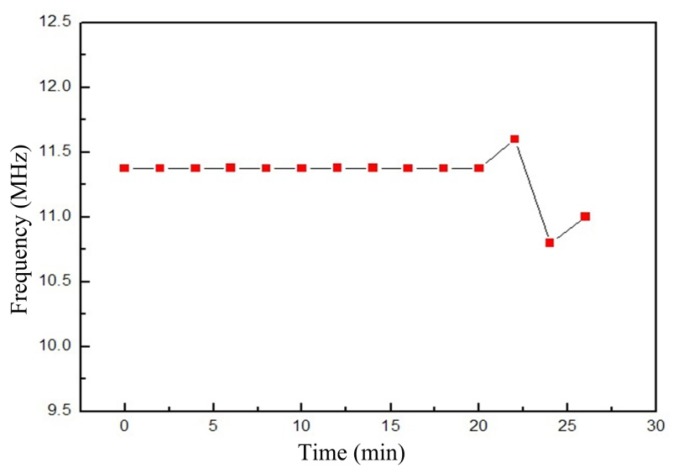
Resonant frequency of the sensor at 850 °C *versus* time.

Figure 10 shows the image of the sensor resonant frequency at 850 °C as a function of time. It is clear that the resonant frequency of the sensor remains stable and drifts only slightly after being at 850 °C for 20 min, and, beyond this range, the frequency of the sensor will not be stable, which verifies the stability and availability of the sensor in high-temperature environments. In order to reduce the frequency of the sensor drift as the temperature increases, the geometric structure parameters of the sensor should be optimized.

### 4.2. High-Thermal-Stress Environments Test

**Figure 11.**
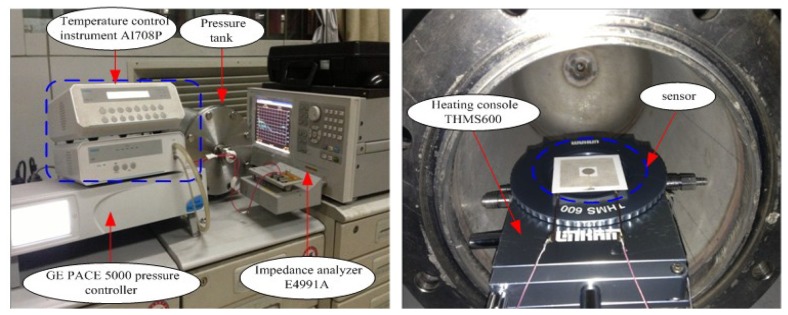
High-temperature-pressure test system image. (**Left**) Entire test setup; (**Right**) Inside the chamber.

After the high-temperature characteristic test, sensor measurements as functions of temperature and pressure are taken using a pressure tank, multifunction THMS600 heating console (Linkam, Surrey, UK) and impedance analyzer E4991A, and the high-temperature-pressure test system platform is illustrated in Figure 11. The pressure can be controlled accurately between the atmospheric pressure and 6 bar using a pressure tank, GE PACE 5000 pressure controller (Druck, Carlsbad, CA, USA), and a nitrogen gas tank. In addition, the temperature can be controlled accurately from room temperature up to 600 °C using the THMS600 heating console and an AI708P temperature control instrument (Yudian, Xiamen, China). The sensor was placed on the heating console surface, which enabled the sensor to operate in a range from 40 °C up to 600 °C. The sensor was connected to the E4991A impedance analyzer used for collecting the resonant frequency signals of the sensor.

**Figure 12.**
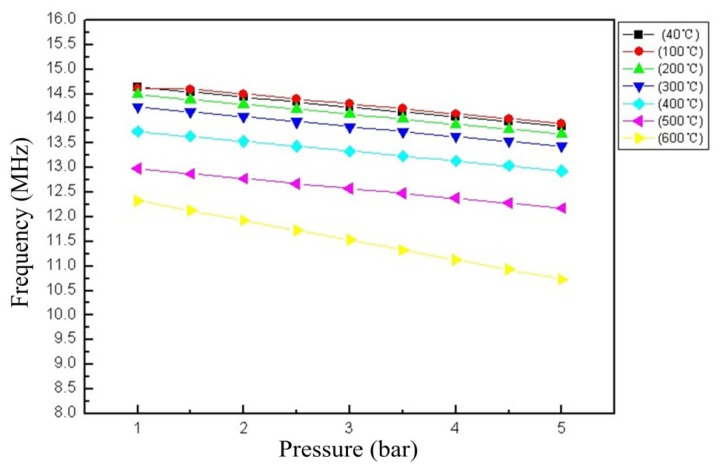
Resonant frequency of the sensor *versus* applied pressure from 1–5 bar as the temperature increases from room temperature to 600 °C.

The sensor was tested in high-temperature-pressure environments, where the temperature was up to 600 °C, and the pressure ranges between 1 and 5 bar. As shown in Figure 12, the stress sensitivity of the sensor is approximately 0.2 MHz·bar^−1^ from 1 to 5 bar between room temperature and 500 °C, which is in agreement with the theoretical calculation values. And, the sensitivity of the sensor is approximately 0.35 MHz·bar^−1^ at 600 °C.

**Figure 13.**
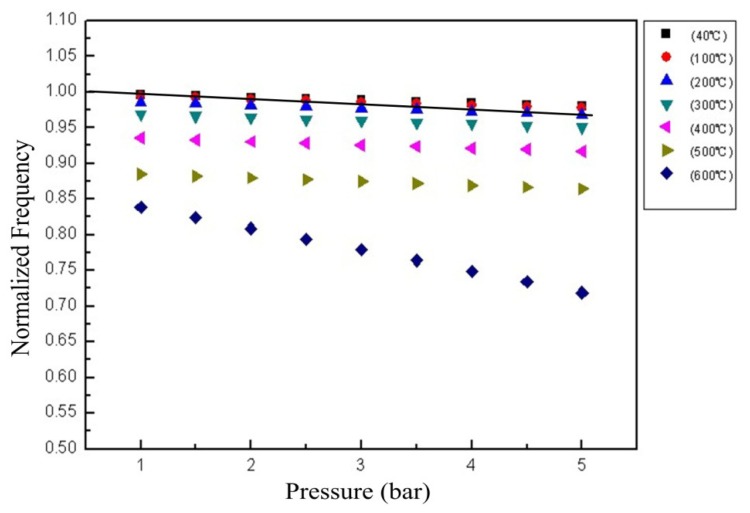
Normalized frequency of the sensor as a function of temperature and pressure.

The comparison between the normalized theoretical and experimental resonance frequencies over the temperature excursion is shown in Figure 13. The resonant frequency of the sensor depends approximately linearly on the pressure at a low temperature. The sensitivity of the sensor from room temperature to 500 °C is relatively stable in the range from 1 to 5 bar, and it will increase above 500 °C. The possible factors that lead to the increase in the sensitivity above 500 °C can be attributed to the change in the properties of the ceramic materials. The permittivity and conductivity of alumina ceramic could increase, as the temperature increase. In addition, the Young's modulus of the ceramic will decrease, as the temperature increase [16].

**Figure 14.**
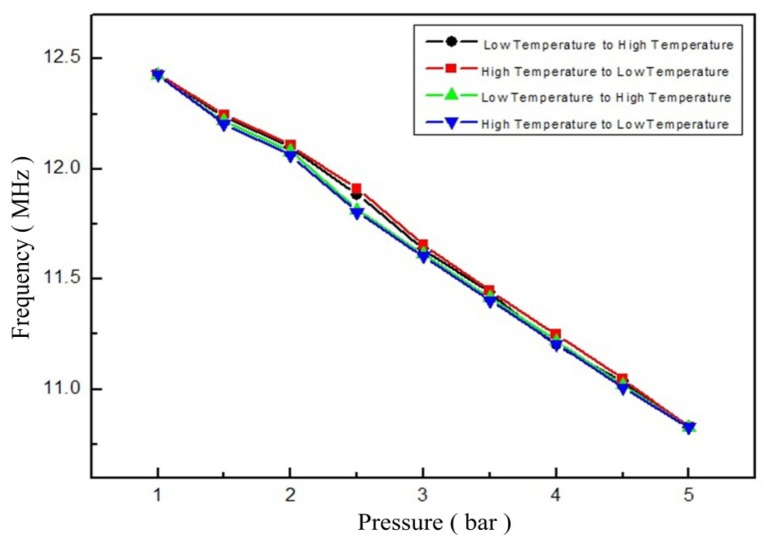
Measured resonant frequency at 600 °C *versus* pressure.

From Figure 14, the sensor exhibits a linear characteristic response between standard atmospheric pressure and 5 bar at 600 °C. The measurement resonant frequency of the sensor gradually reduced, as the pressure increase. And, the repeatability error, hysteresis error, zero-point drift and linearity of the sensor are 8.3%, 5.05%, 1% and 94%, respectively. However, the response time of the sensor is more than 1 s. In future work, the response time of the sensor can be improved by using a new high-temperature material with a lower Young's modulus and optimizing the geometric structure parameters of the sensor.

## 5. Conclusions

Alumina is a critical high-temperature ceramic material used for the fabrication of sensors applied in high-temperature environments. The proposed ceramic sensor, which has a variable capacitance and inductance integrated in the ceramic substrate, is fabricated by a thick-film integrated technology. The high-temperature characterization of the sensor was successfully verified in a high-temperature sintering furnace, which provides evidence that the sensor can work at 850 °C for more than 20 min. In addition, the pressure sensing capabilities of the sensor from 1 to 5 bar at 600 °C are successfully demonstrated in a high-temperature-pressure test setup, where the sensor achieves a linear characteristic response, and the repeatability error, hysteresis error and zero-point drift of the sensor are 8.3%, 5.05% and 1%, respectively. In the future our work will focus on increasing the measurement pressure range of the sensor and improving the sensor sensitivity by using materials with a lower Young's modulus and optimizing the geometric parameters of the variable capacitance and inductance coil of the sensor.

## Figures and Tables

**Table 1.** Geometrical structure parameters of the inductor and capacitor.

**Parameter**	**Symbol**	**Value**
Minimal distance between opposite segments of the inner coil	d_in_	14 mm
Width of conductor	l_w_	0.4 mm
Spacing between adjacent segments	l_s_	0.4 mm
Number of coils	N	15
Radius of one side of cavity	A	6 mm
The thickness of the capacitance sensitive membrane	t_m_	120 μm
The depth of the cavity	t_g_	60 μm

**Table 2.** Sintering process parameters of the silver paste.

**Zone**	**1**	**2**	**3**	**4**	**5**	**6**	**7**
Temperature (°C)	525	680	820	850	850	850	795

**Table 3.** Electrical parameters of the sensor.

**Parameters**	**Symbol**	**Experimental Data**
Inductance	L	∼8.81 μH
Resistance	R	∼10.1 Ω
Capacitance	C	∼13.275 pf
Resonant frequency	F	∼15.28 MHz
Quality factor	Q	∼77
